# Seeing is believing: anti-PD-1/PD-L1 monoclonal antibodies in action for checkpoint blockade tumor immunotherapy

**DOI:** 10.1038/sigtrans.2016.29

**Published:** 2016-11-25

**Authors:** Shuguang Tan, Catherine W-H Zhang, George F Gao

**Affiliations:** 1 CAS Key Laboratory of Pathogenic Microbiology and Immunology, Institute of Microbiology, Chinese Academy of Sciences, Beijing, China; 2 ImmuFuCell Biotechnology Co., Ltd., Beijing, China

## Abstract

Structural immunology, focusing on structures of host immune related molecules, enables the immunologists to see what the molecules look like, and more importantly, how they work together. Antibody-based PD-1/PD-L1 blockade therapy has achieved brilliant successes in clinical applications. The recent breakthrough of the complex structures of checkpoint blockade antibodies with their counterparts, pembrolizumab with PD-1 and avelumab with PD-L1, have made it clear how these monoclonal antibodies compete the binding of PD-1/PD-L1 and function to blockade the receptor-ligand interaction. Herein, we summarize the structural findings of these two reports and look into the future for how this information would facilitate the development of more efficient PD-1/PD-L1 targeting antibodies, small molecule drugs, and other protein or non-protein inhibitors.

The late structural biologist and crystallographer Don C. Wiley once said, ‘I don’t believe the biology until I see it’.^1^ Crystallography (and now cryo-EM) has provided an additional pair of glasses for scientists to see the biological molecules in action.^2^ Three-dimensional structures of molecules have extensively promoted not only the progress of basic biology, but also the development of multiple targeting drugs in industry, for example, influenza A virus neuraminidase (NA) inhibitors and Abl-tyrosine kinase inhibitors.^3–5^ On the basis of the structures of N2 and N9 NAs and the naturally occurring NA inhibitor, 2-deoxy-2,3-didehydro-N-acetylneuraminic acid (Neu5Ac2en), two inhibitors, zanamivir and oseltamivir, were designed and developed to inhibit NA activity and have now been widely used in clinical applications to fight against influenza A virus infections.^3^ These two inhibitors were among the earliest and most successful examples of structure-based drug design and also have led to the development of other anti-influenza inhibitors. Another representative example of structure-based drug design is the tyrosine kinase inhibitor, imatinib mesylate (Gleevec, STI571 or CP57148B), to treat chronic myeloid leukemia as well as other blood neoplasias and solid tumors with etiologies based on activation of these tyrosine kinases.^4^ As an important subfield of structural biology, how will the structural immunology contribute to drug design to modulate immune responses is considered to be a key issue to promote translational medicine in the field.

Immune checkpoint blockade therapy has taken center stage from the corner especially since tumor immunotherapy was selected as Breakthrough of the Year by Science in 2013.^6^ T-cell activation involves multiple paired molecular interactions including T-cell receptor (TCR)/peptide major histocompatibility complex (pMHC) interactions, CD4 (or CD8)/pMHC co-receptor interactions and co-stimulatory ligand-receptor interactions under the current two-signal system theory (Figure 1a left).^7–12^ Besides, activated T cells also need co-stimulatory and co-inhibitory molecules to modulate TCR-mediated antigen specific T-cell responses and self-tolerance.^10,11^ Programmed cell death 1 (PD-1) is a member of the CD28 superfamily and was first discovered as a gene upregulated in a T-cell hybridoma undergoing cell death, therein the name was originated.^13^ PD-1 ligand 1 (PD-L1) and PD-1 ligand 2 (PD-L2) were identified to be the ligands (PD-Ls) of PD-1.^14,15^ Studies show that co-inhibitory molecules such as PD-1 and PD-L1 induce immune suppression in the tumor microenvironment which subsequently leads to the tumor immune escape (Figure 1a middle).^16–19^ Modulating PD-1/PD-L1 paired signal has become the priority choice in immune checkpoint blockade therapy based on substantial evidence indicating that blockade of PD-1 pathway can effectively induce anti-tumor immune responses by restoration of T-cell function and inhibiting intra-tumoral Treg cells within the tumor microenvironment (Figure 1a right).^20–23^ Monoclonal antibodies (MAbs) take advantages of specific binding to antigens with its complementarity-determining region (CDR) loops of both heavy chain (V_H_) and light chain (V_L_) and immune activating mediated by fragment crystallizable (Fc) region (Figure 1b), and thus have been widely used for PD-1/PD-L1 immune checkpoint blockade therapy.^24^ Multiple PD-1/PD-L1 blockade antibodies have been approved for clinical use or have entered into clinical trials, such as pembrolizumab, nivolumab, avelumab and atezolizumab and so on, and have shown great efficacies to treat multiple advanced-stage tumors.^25–28^

Although MAb based immunotherapy has achieved great successes in fighting against multiple tumors in clinical application, some basic questions and concerns yet exist. It is unclear how the MAbs interact with PD-1 or PD-L1 to block the interaction of PD-1/PD-L1, though dominant negative competition for the receptor-ligand interaction is proposed. Are there any hot spots in PD-1 or PD-L1 for checkpoint blockade MAb targeting? Can we predict the possible mutational escapes on PD-1 or PD-L1 under the immune selective pressure of the MAbs during immune checkpoint blockade therapy in the future? Though mutational escape in PD-1 or PD-L1 has not been reported, CD19 mutational escapes or alternative splicing under chimeric antigenic redirected T-cell immunotherapy have already been observed.^29^ And is there any possibility that we could design a better therapeutic MAb targeting the hot spots and avoid antigen mutational escape? All these questions remain unanswered.

The recently reported avelumab/hPD-L1 and pembrolizumab/hPD-1 complex structures by our group and Song group have provided clear structural information on how the therapeutic MAbs abrogate the binding of PD-1/PD-L1.^30–32^ As a PD-L1 targeting antibody, avelumab is a human IgG1 antibody co-developed by Merck (Darmstadt, Germany) and Pfizer, which is now in multiple phase III clinical trials against non-small cell lung cancer (NSLC) (NCT02395172), advanced renal cell cancer (NCT02684006) and gastric cancer (NCT02625610). Complex structure of avelumab/hPD-L1 shows that the avelumab utilizes both V_H_ and V_L_ to bind to the IgV domain of PD-L1 on its side (Figure 1c).^30^ The V_H_ of avelumab dominates the binding to hPD-L1 while V_L_ contributes partial contacts. The binding of avelumab on hPD-L1 predominantly consists of the C, C’, F, and G strands and the CC’ loop of hPD-L1. The blockade binding of avelumab is mainly occupied by the V_H_ chain, with minor contribution from V_L_ chain (Figure 1c). The mechanism of avelumab blockade involves the protruding HCDR2 loop to compete the binding of hPD-1 to hPD-L1. Pembrolizumab (Keytruda, also known as lambrolizumab and MK-3475), a humanized IgG4 monoclonal antibody targeting hPD-1 developed by Merck & Co., Inc., has been approved by the US Food and Drug Administration for advanced melanoma and NSCLC.^24^ Na *et al.*^31^ reported that the interaction of pembrolizumab with hPD-1 is mainly located on the flexible C’D loop and the C, C’ strands of PD-1 (Figure 1d). Though the C’D loop is not involved in the interaction with hPD-L1, it contributes major contacts with pembrolizumab through multiple contacts. Pembrolizumab utilize both heavy chain and light chain to bind to C’D loop of hPD-1. The C’D loop contributed pivotal contacts with pembrolizumab with the fact that one site mutation in C’D loop (D85G) would absolutely eliminate the binding. Thus, the blockade of the hPD-1/hPD-L1 interaction by pembrolizumab is mainly dependent on the binding to the C’D loop and stereo specific blockade on the C and C’ strands of PD-1 to compete with the binding of hPD-L1. The binding affinities (*K*_d_) of pembrolizumab to hPD-1 and avelumab to hPD-L1 are 27.0 and 42.1 pm, respectively.^31^ On the other hand, the binding affinity between hPD-1 and hPD-L1 is 0.77–8.2 μm,^33–35^ which is much weaker than that of the MAbs. The strong binding of pembrolizumab to hPD-1 and avelumab to hPD-L1 would enable the binding priority of the therapeutic MAbs with checkpoint molecules and subsequent blockade of the hPD-1/hPD-L1 interaction.

In addition, there are also very reasons that the development of small molecules or low-molecular weight protein drugs targeting PD-1/PD-L1 signal are in urgent need to achieve a more efficient treatment. First, the efficacy of PD-1/PD-L1 blockade strongly depends on the molecular accession of antibody with PD-1 or PD-L1. However, accession of PD-1 or PD-L1 molecules in the tumor microenvironment by the penetrated MAbs is less efficient because of the large size of the MAbs and the complexity of tumor microenvironments, which further limits their functional potential.^36^ Second, therapeutic MAbs maintained the Fc fragment would induce cytotoxic immune responses even engineered with IgG4 class or antibody-dependent cell-mediated cytotoxicity (ADCC) defective mutations.^37,38^ Because PD-1 and PD-L1 are also expressed on anti-tumor T cells, the utilization with PD-1 or PD-L1 MAbs usually accompanied with undesirable side effect including depletion of the very lymphocytes they are intended to activate. Taken together, development of small molecules or low-molecular weight protein drugs are still in urgent need even with the existence of so many antibody drugs under development or in the market.

Complex structure of PD-1/PD-L1 has brought promising perspectives to structure-based drug design to interrupt the PD-1/PD-L1 interaction. It is now clear how the PD-1 interact with PD-L1 since murine PD-1 (mPD-1)/hPD-L1, mPD-1/mPD-L2 and hPD-1/hPD-L1 complex structures were resolved.^39–41^ Based on the complex structure of mPD-1/hPD-L1, Roy Maute *et al.*^42^ designed a yeast-surface display system for a directed evolution to generate high-affinity PD-1 that antagonizes PD-L1. 22 amino acid residues of PD-1 contributing to the contacts with PD-L1 were selected for randomization. Two-generations of yeast-surface display library evolution were conducted which resulted in a high-affinity consensus hPD-1 with enhanced binding affinities (*K*_d_) of ∼100 pm with hPD-L1 compared with the mild binding affinities of a *K*_d_ value of 3.8 μm for wild type hPD-1. In contrast to anti-PD-L1 MAbs, high-affinity PD-1 enabled superior tumor penetration without inducing depletion of peripheral effector T cells. In addition, the high-affinity PD-1 also showed superior tumor suppression efficacy compared with antibodies in mouse colon carcinoma model. Moreover, a small molecule drug developed by Curis, Inc. (Lexington, KY, USA), CA-170 (oral PD-L1, PD-L2 and V-domain Ig suppressor of T-cell activation (VISTA) checkpoint antagonist), has entered into Phase I clinical trial in patients with advanced tumors and lymphomas (NCT02812875).

The recent report on pembrolizumab/hPD-1 and avelumab/hPD-L1 complex structures have enabled the scientists to see how the checkpoint blockade MAbs interact with the corresponding targets and how this would compete the binding with PD-1/PD-L1. All this information would shed light on the development of the next-generation MAbs or small inhibitory molecules. However, additional therapeutic antibody/PD-1 (or PD-L1) complex structures are still needed to draw a clearer map of how therapeutic MAbs work. Structural immunology has now helped the scientists to see what the immune checkpoint therapeutic MAbs look like and how they work, it is now the responsibility of researchers from both basic science and industry to build more efficient therapeutics to bedside.

## Figures and Tables

**Figure 1:**
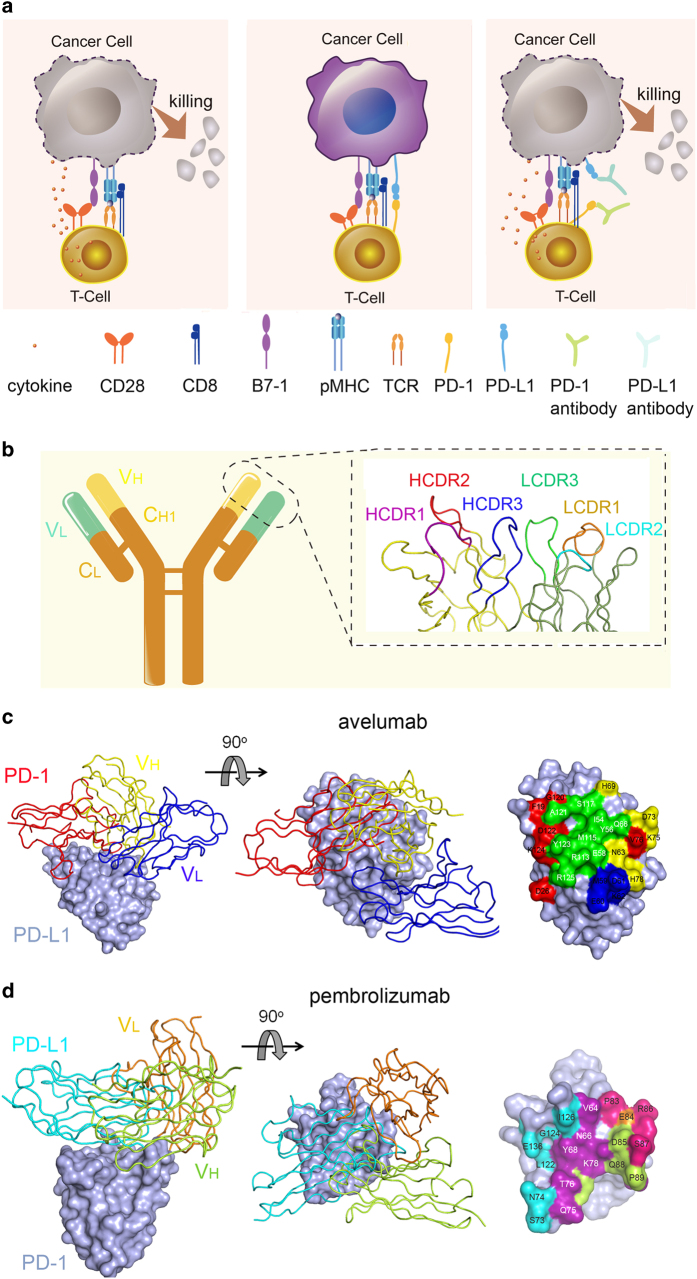
Monoclonal antibody-based immune checkpoint blockade and tumor immunotherapy. (**a**) Mechanisms of antibody-based immune checkpoint blockade for tumor therapy. Left, tumor specific T cells could kill the targeted cancer cells through the interaction of specific TCR, co-receptor CD8 and co-stimulatory molecules on T cells and the cancer specific antigens presented on cancer cells. Middle, upregulation of PD-1 on T cells and PD-L1 on cancer cells would induce the exhaustion of anti-tumor T cells and subsequent tumor immune escape. Right, monoclonal antibodies with PD-1/PD-L1 blockade activity would restore tumor specific T-cell function and kill the tumor cells. (**b**) Diagrammatic sketch of monoclonal antibody. The fragment of antigen binding (Fab) of monoclonal antibody consists variable region of both heavy chain (V_H_) and light chain^25^ and consistent region of heavy chain (C_H_1) and light chain (C_L_). The specific binding to antigen usually involves the three CDR loops from both V_H_ and V_L_. (**c**) Superimposition of the hPD-L1/avelumab complex structure with the hPD-1/hPD-L1 complex structure. hPD-1 and avelumab are shown as ribbon (hPD-1 in red, avelumab-scFv V_H_ in yellow, and V_L_ in blue) while hPD-L1 was shown in surface mode. Right, binding surface of hPD-L1 for hPD-1 or avelumab. The binding residues for hPD-1 on hPD-L1 are colored in red, whereas residues contacted by the avelumab V_H_ or V_L_ are colored in yellow or blue, respectively, and the overlapping residues used by both the receptor hPD-1 and avelumab are colored in green. (**d**) Superimposition of the hPD-1/pembrolizumab-Fab complex structure with the hPD-1/hPD-L1 complex structure. Left, hPD-L1 and pembrolizumab are shown as ribbon (hPD-L1 in cyan, pembrolizumab V_H_ in lemon, and V_L_ in orange) while hPD-1 was shown in surface mode. Right, binding surface of hPD-1 for hPD-L1 or pembrolizumab. The binding residues for hPD-L1 on hPD-1 are colored in cyan, whereas residues contacted by the pembrolizumab V_H_ or V_L_ are colored in lemon or orange, respectively, and the residues that contacts with both V_H_ and V_L_ are colored in hotpink. The overlapping residues used by both hPD-L1 and pembrolizumab are colored in purple.
